# Nanopesticides in Agriculture: Benefits and Challenge in Agricultural Productivity, Toxicological Risks to Human Health and Environment

**DOI:** 10.3390/toxics9060131

**Published:** 2021-06-04

**Authors:** Marco Chaud, Eliana B. Souto, Aleksandra Zielinska, Patricia Severino, Fernando Batain, Jose Oliveira-Junior, Thais Alves

**Affiliations:** 1 Laboratory of Biomaterials and Nanotechnology—LaBNUS, University of Sorocaba, Sorocaba 18078-005, Brazil; fbatain@gmail.com (F.B.); thaisfrancinealves1@gmail.com (T.A.); 2 Technological and Environmental Processes, University of Sorocaba, Sorocaba 18023-000, Brazil; jose.martins@prof.uniso.br; 3 CEB—Centre of Biological Engineering, University of Minho, Campus de Gualtar, 4710-057 Braga, Portugal; 4 Department of Pharmaceutical Technology, Faculty of Pharmacy, University of Coimbra, Pólo das Ciências da Saúde, Azinhaga de Santa Comba, 3000-548 Coimbra, Portugal; 5 Institute of Human Genetics, Polish Academy of Sciences, Strzeszyńska 32, 60-479 Poznań, Poland; zielinska.aleksandra@wp.pl; 6 Institute of Technology and Research—ITP, Nanomedicine and Nanotechnology Laboratory (LNMed), Aracaju 49010-390, Brazil; pattypharma@gmail.br; 7 Laboratory of Applied Physics Nuclear—LAFINAU, University of Sorocaba, Sorocaba 18023-000, Brazil

**Keywords:** nanopesticides, agrochemical ingredients, controlled release, toxicological risk, environmental risk, pesticides, agricultural productivity, stimuli-responsive nanoparticles

## Abstract

Nanopesticides are nanostructures with two to three dimensions between 1 to 200 nm, used to carry agrochemical ingredients (AcI). Because of their unique properties, the loading of AcI into nanoparticles offers benefits when compared to free pesticides. However, with the fast development of new engineered nanoparticles for pests’ control, a new type of environmental waste is being produced. This paper describes the nanopesticides sources, the harmful environmental and health effects arising from pesticide exposure. The potential ameliorative impact of nanoparticles on agricultural productivity and ecosystem challenges are extensively discussed. Strategies for controlled release and stimuli-responsive systems for slow, sustained, and targeted AcI and genetic material delivery are reported. Special attention to different nanoparticles source, the environmental behavior of nanopesticides in the crop setting, and the most recent advancements and nanopesticides representative research from experimental results are revised. This review also addresses some issues and concerns in developing, formulating and toxicity pesticide products for environmentally friendly and sustainable agriculture.

## 1. Introduction

The worldwide population growth demands access to quality food capable of meeting people’s needs on all continents. Associated with the pressure of economic investment in agricultural commodities, we have also witnessed the increased use of fertilizers, insecticides, herbicides, and other agrochemicals to improve agricultural productivity. Nevertheless, farmers, food producers, financing agencies and governments must be committed to balancing the benefits and risks of cyclical declines generated by the indiscriminate use of agrochemical technologies in environments, ecosystems, consumers and all the productive sector involved directly or indirectly in the agricultural chain [1].

Worldwide, countless international patents and licensed products link farming peculiar technology and agriculture, focusing on providing several commodities like food, fuel, and wood for humankind [2].

Agrochemical technology is typically aimed to protect crop areas against pests (pathogens, harmful insects, parasitic weeds) that compromise production and productivity, including diagnosing soil and plantations vitality, livestock, and fishery products control. Nevertheless, the indiscriminate use of pesticides applied against dangerous pests and insects has also been adversely affected production, inducing resistance to pathogens and insects, rising demand for new agrochemical and increasing the environmental imbalance [3].

The higher total surface area occupied by agrochemicals nanoparticulates offers overall greater contact with crop pests, making them as efficient as they are unpredictable. The unreasonable and unsystematic use of agrichemical intensifies pathogen resistance, reducing nitrogen fixation and biodiversity, and increasing bioaccumulation of pesticides in agricultural, livestock goods, and in organisms of the water environment, which poses a severe and progressive threat both to the ecosystem and to humans and an obstacle against the development of sustainable agriculture [4,5].

In this review, the primary forms of presentation and composition of innovative-nanopesticides are discussed, together with the pros and cons of nanomaterials in controlling agricultural pests, environmental risks and human and animal health effects. Special attention is given to the main nanomaterials used in the agrochemical industry, commonly focused on laying down the crop, regardless of their safety profile.

## 2. Nanotechnology

Remarkable opportunities to renew agriculture practices have been introduced by using nanotechnology-based delivery systems, attributed to the smart controlled release profile of fertilizers and agrichemicals require to enhance crop productivity [6]. Such systems play a critical role in agriculture, improving fertilizers and agrochemicals performances [7]. Although the future of nanopesticides in agriculture development appears promising, the human exposure to dangerous agrochemicals able to cross biological barriers (e.g., blood-brain barrier, blood-placental barrier, and blood-retinal barrier) is a significant concern as it can cause irreversible damage to vital organs. The risks posed by the exposure to hazardous nanopesticides, which are able to induce toxic and genotoxic events, are currently receiving great attention by studying the effect not only on the chemical composition of the bulk material but also on the physicochemical properties of nanopesticides such as size, electrical charge, and surface properties [8,9].

Innovative-nanopesticides are nanomaterials engineered to plant protection, minimise application losses, increase coverage on the leaf, enhance stability, and reduce the quantities of formulation’s ingredients. Nanopesticides formulations can be divided into self-organised systems like liposome, dendrimers, metallic and bimetallic nanoparticles, and active encapsulating ingredients like nanoemulsion, polymeric nanoparticles, lipide nanoparticles and nanotubes.

### 2.1. Nanopesticides

Nanopesticides stand for pesticides formulated in nanomaterials to find applications in the agricultural field, whether specially fixed on a hybrid substrate, encapsulated in a matrix or functionalized nanocarriers for external stimuli or enzyme-mediated triggers. Nanosized particles, coupled with their shape and special properties, are thought to explore pesticide activities in nanocarrier innovative formulations based on several materials like silica, lipids, polymers, copolymers, ceramic, metal, carbon and others [10].

The nanopesticide formulations can increase water solubility, bioavailability and protect agrochemicals against environmental degradation, revolutionizing the control of pathogens, weeds, and insects in the crops [2]. However, the nanomaterial features are also borderline their cytotoxicity and genotoxicity.

The indiscriminate and irrational use of pesticides influence the balance of the ecosystem and expose everyone’s health to risk. Adverse effects of short-term (acute) and long-term (chronic) resultant of occupational or accidental ingestion of pesticide residues from food, water-drinking is fatal or disability-adjusted life years (DALY). Children are more vulnerable to pesticide exposure and are subject to permanent tissue and organ damage. Between them, the centra and peripherical neurotoxicity and the effect on the loss of blood ability for coagulation are meaningful reasons for concerns [11]. Indeed, a detailed assessment of the pros and cons that influence the activity and toxicity of nanopesticides is crucial for the safe and sustainable development of the already approved use of nanoparticles in agriculture.

The effect of formulations on the behavior of nanopesticides in the environments, ecosystems, farmer workers, consumers, and all productive sector involved in the agriculture chain is not entirely known [12]. However, the critical role of nanoformulations in reducing the active ingredient’s degradation, improving water solubility equilibrium, and increasing the biological availability of actives ingredients are known. Specifically, to avoid endemic infestation of pests, plant injury and economic loss by decreasing the quality and quantity of agricultural products and foods [13].

Nanopesticides from runoff of agricultural and industrial wastewater during the precipitation event by soil permeating leaching phenomenon reach the water supply, affecting its quality, increasing the human exposure time and concerns for the ecosystem. It has been noticed that nanoparticles could cause toxicological effects by their biomimetics properties and high ability of distribution and bioaccumulation in soil, water environments, foods, and consequently in all animals, especially in mammals [3,4,5,14]. For humans, the range of side effects related to individual susceptibility and exposure time to nanoparticles leads to acute and chronic pathological manifestations that include the systems respiratory as cardiovascular, lymphatic, autoimmune, neurologic, and various cancers that can manifest instantly following exposure or many years later as a result of bioaccumulation [15] and unique nanoparticles properties [11,16,17].

Modified-release nanopesticides can be categorized into two groups: pesticides that are chemically linked, and others, which are physically incorporated formulation of the pesticide, activated at once after delivery in agriculture (Table 1).

### 2.2. Innovative-Nanoformulation Encapsulating Pesticides

Encapsulation is a process of surrounding one biologically active ingredient with the intention that the core confined material or into capsule walls can be released to the environment under specific conditions over a predetermined time or when external stimuli activate the capsule walls to break, melt or dissolve slowly. In the formulation, the active ingredient into nanocapsule is chemically bound or physically adsorbed in a matrix by different techniques, to be later released by chemical bonds cleavage or by physical diffusion.

There are distinct reasons for nanoencapsulation of pesticides between them to combat loss of efficacy due to evaporation, degradation and leaching, and increased activity due to better interaction with the pathogen, insects, weeds, and other pests. However, there also are reasons to consider the pros and cons of using pesticides in nonencapsulated systems. If, on the one hand, the agrochemical companies’ current focus is on laying down the crop regardless of the best condition, on the other hand, up-to-date research about innovative materials systems stimuli-responsive at the light, pH, temperature, enzyme, and others has been reported for agrochemical extending release, targeting delivery, decreasing the usage amount, and reducing leaching and drift, and improving the utilization efficiency of the pesticide [34,43,46,47].

Innovative and controlled release formulations (Table 2) are described as depot systems; this means that there will be a delay between the delivery time of encapsulated nanopesticide in the crop and the start of the AcI release process, which is different from a conventional formulation in which a burst of AcI release will occur at once after the delivery of formulation in the crop. Therefore, either for the depot system or immediate another, the desired effect will only be noted when the minimum effective concentration is achieved [48]. Under depot system conditions, a very high loading rate benefits are controlled release for an extended period and reduction dosing frequency to every five months. In addition to prolonging the activity of the nanopesticides, the required application amounts are several orders of magnitude lesser than the conventional formulation [49].

Innovative nanotechnologies aim to reduce the indiscriminate and abusive use of conventional pesticides and ensure a safe application. Grafted target nanoparticle formulations for environmental stimuli-responsive are currently the uppermost technological advance for the safe use of pesticides and new ways to provide nanopesticides innovative material. 

Nanostructured matrix systems such as nanocapsules, nanospheres, nanovesicles have been designed and functionalized using sensitive polymer material to deliver pesticides [12,34,52]. A submicron capsule of Seltima^®^, to address pyraclostrobin’s toxicity, allowed to build a controlled release system based on the humidity sensitivity, purposefully, for the rice leaves and other crops without harming the aquatic environment. Thermo-sensitive and efficiency-controlled release time of pyraclostrobin nanocapsule obtained by emulsion polymerization using poly(*N*-isopropyl acrylamide-co-butyl methacrylate) improved stability and action time of pesticides [48,50].

**Figure 1:**
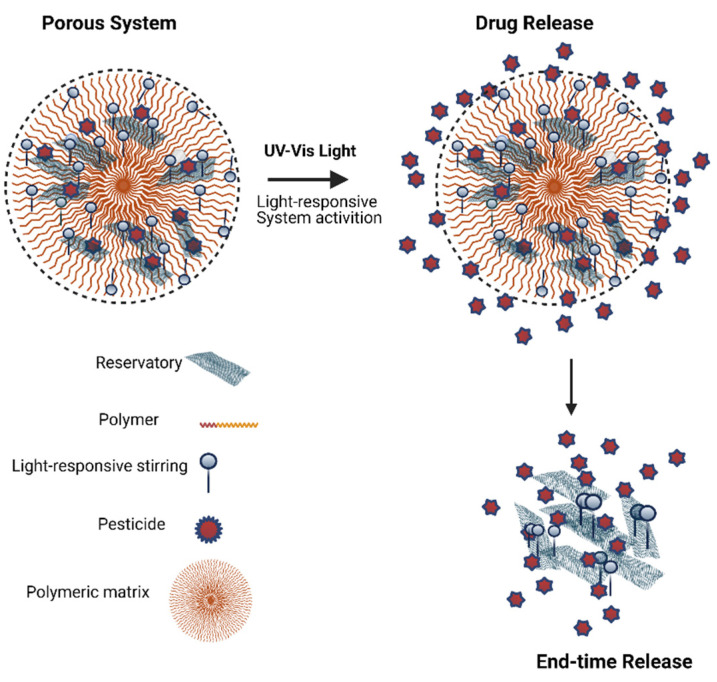
Schematic illustration of light-responsive system for modified pesticide release.

A stimuli light-responsive controlled release (Figure 1) has been used as a delivery system in various agro-livestock application areas with environment-friendly property in the field and greenhouse cultivation. Poly(ethylene oxide-b-methacrylic acid) (PEO-PMAA), a light-responsive co-polymer, was obtained with light-sensitive groups; after self-assembly and encapsulation of pesticide, the controlled release has achieved, and an extensive residual activity of the formulation was observed [12]. 

**Figure 2:**
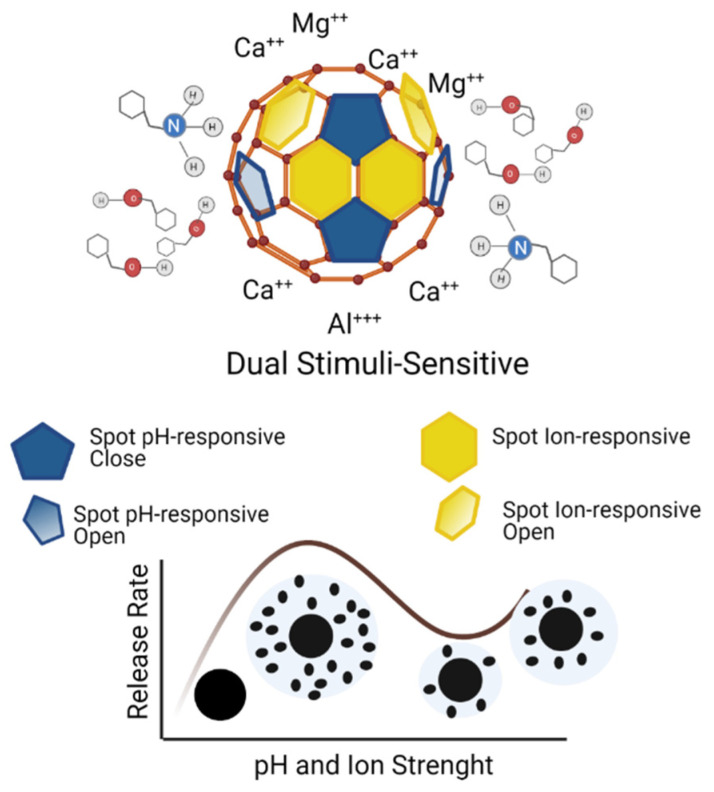
Schematic illustration for dual stimuli-responsive and diffusion release profile.

A dual responsive system for pH and ion strength (Figure 2) is an engineering effort focusing on innovative material to reduce the amount of pesticide used in the crop, innovative modified-release in a complex environment, decreased pesticide leaching and decomposition, and the minimal adverse impact ecosystem [55]. An innovative release system for environmental stimuli can prevent the premature complexation of agrochemicals, inhibit the sulfidation reaction, and exhibit extended pest control capabilities [10,47].

## 3. Physical and Chemical Systems for Agrochemical Delivery

Carbon nanotubes single-walled or multi-walled (MWCNT) as innovative nanomaterials have interesting rising application in agriculture as a slow-release system of fertilizer and pesticide and as a biosensor of the AcI. The positive effect of carbon nanotubes in the enhanced plant biomass, length of root and shoot, and seed germination have reported [18]. Although the mancozeb antifungal pesticide encapsulated into hybrid material (MWCNT-graft-poly-citric acid) nanoparticulated showed more stability and effectiveness than bulk pesticide and increased water solubility of AcI, a contradictory effect of carbon nanotubes has been noticed. The carbon nanotubes can cause negative outcome on plants leading to cell death and a decrease in soil microbial population due to reactive oxygen species (ROS) forming [53].

Proteins and peptides such as scorpion and insect toxins, snail poison and hormones have multifunctional properties and insecticidal activity. However, the facile metabolism of proteins or peptide in the insect’s digestive system is the major limitation for applying insecticide peptide-based. This protease degradation mechanism in the digestive system and hemolymph and the movement across the midgut ventriculus in insect pests are the most significant challenge for pesticide activity. These barriers impact food production and increase the limit of insects’ resistance to pyrethroids, organophosphates, organochlorines, and carbamates [56].

To overcome physical, chemical and biological barriers is the goal of the novel and smart nanopesticides. A strategy of developing AcI release systems for insecticide effect including hydrophilic polymer conjugated to TMOF (trypsin modulating oostatic factor) increased stability and inhibited metabolism. It enhanced the bioaccumulation insecticide across the insect gut lumen in the hemolymph (*Spodoptera frugiperda*), inhibiting oocyte maturation and reducing insect reproduction [19,22,56].

Particles based on ionotropic reactions have gained interest as pesticides nanocarrier due to low cost, production being easy under mild conditions, and a GRAS medium, size control, biocompatibility, nontoxicity, and variety of crosslinking agent [57,58]. Sodium alginate and hydrosoluble calcium chloride were used to prepare loaded nanoparticles cypermethrin with sustained-release effect. The cypermethrin entrapment efficiency and loading were 96% and 78%, respectively, with cypermethrin molecules occupying the nanoparticles’ inner side. Cypermethrin loaded Ca-alginate nanoparticles reached better results than direct application to plants in soil pot experiments [20].

**Figure 3:**
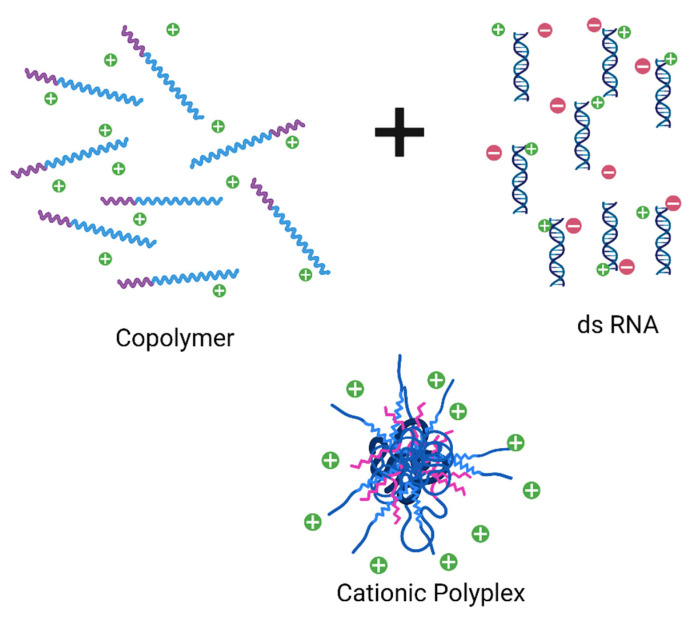
Schematic illustration of self-assembly reaction between cationic copolymer and dsRNA (double-stranded RNA) to prepare a cationic polyplex system as pesticide reservoir pH-sensitive.

Polyelectrolyte complexes as nanopesticide carriers have attracted the productive and rural producers’ attention by reducing the recommended dose/Ha and by reducing herbicide and agrochemicals with a high leaching rate in the soil. Clay-gelatine based formulation of the MCPA herbicide reduced water environmental risks compared with the conventional formulation of MCPA.K-salt in solution water [21]. The polyelectrolyte complex system has also been used as a biosensor in electroanalytical chemistry to detect food poisoning. The chitosan–pectin polyelectrolyte complex was used for electro-reductive determination of metribuzin (pesticide) and metronidazole (antibiotic) without any metallic compound [59]. Besides, the interpolyelectrolyte complex (IPEC) system (Figure 3) is a cationic polyplex, easily redispersed in water, that can condense genetic material until colloidal size (50–500 nm). The release test showed that IPEC nanodispersions behave as a pesticide reservoir, showing a controlled release rate that significantly increased or decreased in physiological fluids by ionic exchange pH-based [60].

RNAi-based technologies have designed for the introduction of dsRNA in insects’ crop for pest control. The dsRNA, when processed into siRNA effectors, inhibited the gene expression of *Spodoptera frugiperda*. A biomimetic cationic polymer like pGMPA was synthesized with dsRNA by reversible addition−fragmentation chain transfer (α-RAFT) polymerization to obtain an interpolyelectrolyte complex that had efficiently taken up by cells and functioned as a trigger for RNAi. This strategy was reported as efficient to deactivate the gene in a multi-drug-resistant insect [22].

Metallic nanoparticles are made on a single-phase reduction of the distinct metal precursors in an aqueous medium. On account of known surface plasmon resonance characteristics, these nanoparticles have unique insecticide properties. The noble metal ions like Ag, Au, Cu, and others are chemically adsorbed to the pathogen and insect surface by functional groups on the cell wall polysaccharides, non-specific bacterial toxicity mechanisms an alternative to traditional pesticides to overcome bacterial resistance [61].

Physical systems for drug delivery are more friendly than chemical systems. Applying these systems for nanoencapsulation of biocide and fertilizer has been used in agriculture to improve agrochemicals’ efficiency, safety, and stability for a more extended period. Agrochemicals encapsulate in the physical systems are referred to as internal or filler material entrapped in nanoparticles. In this case, it is consensus to refer to the term nano when nanoparticles are higher than 100 nm. Materials like clay minerals (bentonite, smectite, chaolite, montmorillonite), LDHs anionic clay (layered double hydroxides), lipid (waxes, triglycerides, fatty acid, surfactants), inorganic porous (silica, ceramic, polytriphenylamine), natural polymers (cellulose, starch, gelatin, albumin, chitosan), synthetic polymers (polyamidoamine, polyethylene oxide, polylactide, cyanoacrylates), among others, have been used for nanoencapsulation in systems classified like physical [10,21,28,29,62]. Agrochemicals encapsulate in the physical systems are referred to as internal or filler material entrapped in nanoparticles. In this case, it is consensus to refer to the term nano when nanoparticles are higher than 100 nm. Materials like clay minerals (bentonite, smectite, chaolite, montmorillonite), LDHs anionic clay, lipid (waxes, triglycerides, fatty acid, surfactants), inorganic porous (silica, ceramic, polytriphenylamine), natural polymers (cellulose, starch, gelatine, albumin, chitosan), synthetic polymers (polyamidoamine, polyethylene oxide, polylactide, cyanoacrylates), among others, have been used for nanoencapsulation in systems classified like physical [10,21,28,29,62].

Clay and LDHs-based nanoparticles are effective for protecting the pesticides against volatilization and photo-degradation. The mineral clay nanoformulations are recognized as safer to the ecosystem due to reduced drift and leaching rates than other carrier systems. The chemical property of these nanocarriers is attributed to the reversible binding of the pesticide with the matrix. [21,30].

A light-responsive controlled-release pesticides nanoparticle was fabricated using attapulgite and biochar to form a porous system coated with amino silicon oil and grafted with azobenzene. The composites environmentally friendly and high adsorption ability role as an adsorbent for glyphosate. The release behavior in water was studied under UV-VIS light (365–435 nm). The core-shell nanostructures showed an efficient stimuli-responsive controlled by UV-VIS light, adhesion on weeds leaves reducing the glyphosate loss and a notable increase in herbicides efficiency [35,43,46].

**Figure 4:**
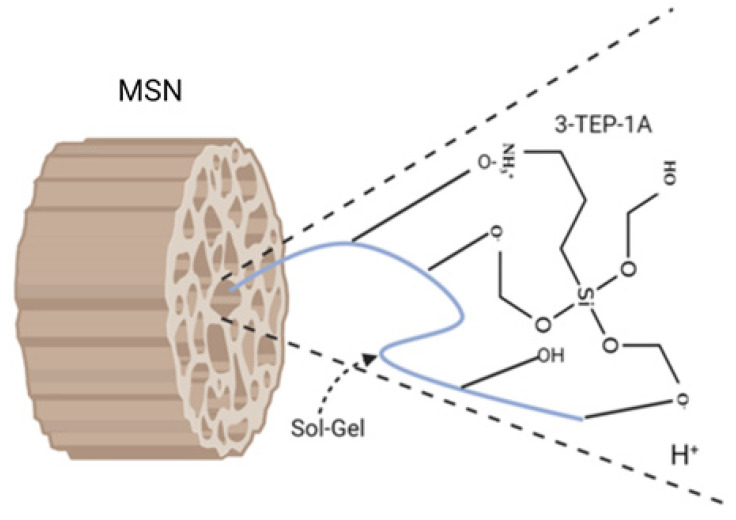
Schematic illustration of the sol-gel interaction pH-responsive and anchoring the 3-(triethoxysilyl) propane-1-Amine (3-TEP-1A) molecule on the inner pore surface of the modified mesoporous silica nanoparticles (MSN).

The sol-gel composite process begins when an organic molecule is entangled in a silica-based matrix’s inner porosity by the dopant agent’s addition (Figure 4). Organically modified silica nanoparticles have the versatility of organic polymers and other advantages due to the sol-gel process, such as tail shape, density, and surface property unique. These exceptional properties supply a far higher load of active ingredient that can be entangled (up to 90% wt.), control over the sustained release rate (up to months) and enhance stability even in unfriendly environmental conditions [41].

Currently, sol-gel composite is an innovative delivery nanocarrier for pesticides that can be stimuli-responsive. The azoxystrobin loaded mesoporous silica nanoparticles change surface with carboxymethyl-chitosan was explored as a sustainable fungicide in the tomato plant. The results showed an increase in the loading content, controlled release driven by pH and better fungicidal effect [42].

The development of biopesticides as an alternative approach to chemical control measures has gained distinction due to precision, the species-specific target organism, low toxicity, and a strategy to overcome multidrug resistance barriers in pathogens. Genetic material delivery in the nonencapsulated system is a novel and remarkable strategy for controlling pests and plant protection against insects due to sequence-specific endogenous RNAi. Currently, it has used to control pests, with or without the trigger of ds RNA [63].

pGPMA solutions were prepared to complex 1.0 μg dsRNA via co-precipitation to form IPEC to trigger RNAi in insensitive insect crop pests. The results demonstrated a gene suppression by RNAi *per os* in an insensitive insect species [22]. Ladybird beetles (*A. bipunctata* and *C. septempunctata*) were selected to evaluate the dietary RNAi response and effects of ingesting dsRNA. The results have disclosed that *C. septempunctata* more sensitive to the RNAi triggered; however, the reason for the different effects of insect resistance is unclear [64]. Nevertheless, it has been reported that species genetically similar and closely susceptible to the environment for RNAi have similar susceptibility [63].

## 4. Lipid-Based Nanopesticides

The employing of specific lipids and surface modifier chemical agents for engineering the nanoemulsion, micellar and vesicular systems can be designed and prepared for target delivery pesticide based on the environmentally responsive controlled release. Nanoemulsified carriers for the release of agrochemicals are the subject of intense research. Given the potential of free energy, the large surface area, unique chemical properties, and biocompatibility of the emulsified systems, several nanopesticide emulsion-based types have emerged to improve the use efficiency and modulate the pesticide release profile. All formulations emulsion-based are designed to enhance active compounds’ solubility, improve bioavailability, stability, and wettability, resulting in better pest and weed control and contributing to the advancement of up technology in agrochemicals delivery the agricultural sector [36,65].

**Figure 5:**
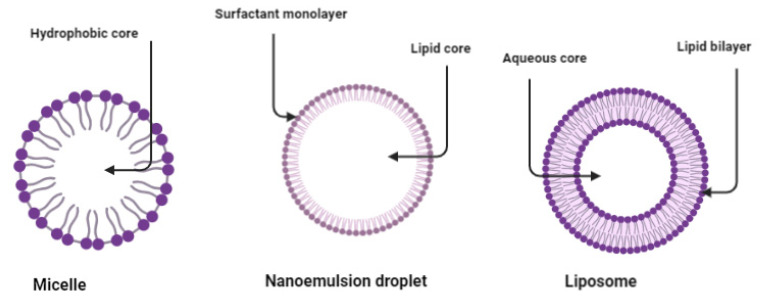
Structural form and composition of the micelle, nanoemulsion droplet and liposome formulations. The tail of the surfactant forms the hydrophobic core of the micelle. Nanoemulsion water-in-oil stabilized by surfactant surround lipid core. Liposome lipid bilayer and aqueous core.

**Figure 6:**
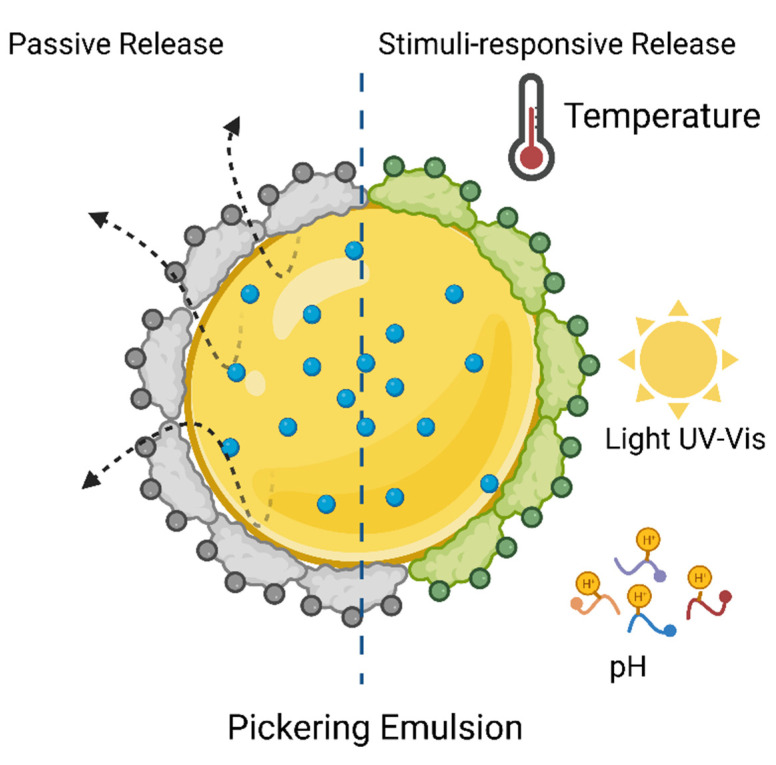
Structural form of Pickering emulsion and release mechanisms passive and stimuli-responsive.

Figure 5 and Figure 6 display a schematic illustration of colloidal systems emulsion-based used as a carrier of pesticides and doped with stimuli-responsive agents such as thermal, light and pH. The micelle’s formation or the so-called micellization is a supramolecular nanoparticle assembly of spontaneous formation, commonly reported as a cluster spherical of tiny micelles attributed to auto-aggregation that occurs spontaneously during the synthesis process [34,66].

An amphiphilic polymers composite was synthesized, and the nanoscale mixed supramolecular cooperative assembly behavior was evaluated in the pyrethrin presence. The formulation supplied a temperature-sensitivity modified-release mode that regulated the pyrethrin delivery rate due to structural deformation of copolymer temperature-induced. This technology showed three key advantages: photostability, enhanced larvicide action under natural conditions, and sustainable release [34].

The Pickering emulsions (PE) are lipid colloidal dispersions without surfactants, physically stabilized by polymer particles or inorganic particles with good wettability, and they have been widely used in pesticides [67] as release stimuli-responsive systems based on the ability to transform stable and unstable form (Figure 6) through pH, temperature, and humidity [68]. Anisotropic silica nanoparticles alginate grafted has prepared, and the effect of structure and pH-responsive on the properties of PE as a carrier for λ-cyhalothrin have investigated their application in modified release systems pH triggered. The results showed that PE’s stability enhanced with viscosity and increased surface charge of the nanoparticles. The release profile is compatible with sustained-release systems triggered by pH from 2.0 to 9.0, at 240 min, cumulative λ-cyhalothrin release from pH 4 reached 87%, 28% (pH 2) and 53% (pH 9) [35].

Nanoemulsions based on lipid mix has better efficacy compared to conventional pesticides. *A. indica* (neem oil) and *C. nardus* (citronella oil) are known for having pest control properties. However, the use of these oils and others recognized as biopesticides are limited by poor water solubility. An approach to overcome this shortcoming is by nanoencapsulation of the oils in the nanoemulsions system. The encapsulation and effect of neem oil and citronella oil using spontaneous emulsification technique have assessed against fungi. The results showed in vitro fungicide activity and a meaningful way to transport hydrophobic pesticides to control plant disease caused by fungus. However, these nanoemulsions prepared with the own oil need to be evaluated in environmental conditions [37].

Liquid crystals are among the most relevant pharmaceutical innovation design to enhanced water solubility, efficacy, stability, and safety of active AcI. Self-assembly nanostructure systems such as lyotropic liquid crystal have properties unique due to the structural ordering or as complex 3D-network forming lamellas, whose thermodynamic properties allow biological activity at the molecular level [69]. Liquid crystal enhancing tissue penetration of the active AcI and increase bioadhesion to the leaf, increasing efficiency and decreasing the environmental damage. Phytantriol was formulated in lyotropic liquid crystal and evaluated for structural equilibrium. The results have shown that Phytantriol in the emulsion form interacts with lyotropic liquid crystal combined with pesticides used as adjuvants, which revealed a potential unlimited to transport hydrophobic herbicides [38].

Liposomes as nanocarriers are vesicle-like matrix systems based on biodegradables and biocompatible materials and designed for active compound modified-release. When engineered, the liposome as a pesticides nanocarriers can have the surface functionalized by specific compounds, and properties such as lipophilicity, hydrophilicity, targeting, flexible wall, release controlled, and biodistribution has been reported. Besides, the liposome surface’s functionalization can be engineered to preserve active AcI against photodegradation and thermal degradation [12,70,71]. Liposome as a nanocarrier for α-cypermethrin and etofenprox was prepared and functionalized with chitosan to change intrinsic surface charge, increase the thick, make a depot system, and gets a more extended period for delivery of active AcI [39].

## 5. Nanocarriers and Toxicity of Pesticides

Despite nanotechnology’s progress in plant science, the comprehension of nanomaterials-plant-environmental interactions, including bioavailability, bioaccumulation, drift, toxicity, and agro-ecosystem safety, is still insufficient to measure the pros and cons of AcI nanotechnology in the crops [72]. Nanopesticides are currently involved in the reformulation of registered AcI, aiming to improve performance compared to the existing non-technological AcI and counterbalancing current agrochemical products’ disadvantages. Understanding the plant response to engineering nanopesticides exposure and correct assessment about toxicity depends on a deep study addressing some bottlenecks and aspects still not fully exploited.

The nanocarriers are among the strategies most target to overcome significant modern farming challenges, especially those related to increasing production vs environmental impacts and pesticides reduced application rates [1,6,73]. According to earlier studies, half of the applied pesticides pervades various microhabitats near crops that contaminate the water table, rivers, and lakes, whether through leaching or air stream. However, reaching all productive agriculture goals without complete comprehension of challenges seems to enhance the risks when AcI and nanoparticles are more harmful to health and the ecosystem [74].

The target and controlled release of nanopesticides based on the trigger are desirable for reaching the pests’ effective activity and reducing environmental pollution, reducing the bioaccumulation effect and threatens ecosystems and human health. Over the past few years, nanotechnology dealing with toxic effects and risks associated with nanoparticles, anionic nanoparticles, surfactants, solvents, wetting, and agents inducing appeared from understanding toxicity and genotoxicity of the nanoparticles in humans. However, the toxicological risk of reaching all goals seems to enhance when AcI and nanotechnology are associated [2,36].

Materials used in the nanoparticle’s synthesis can produce a toxic effect in plant, human and other vertebrate classes [75]. Due to their biomimetic properties with the cellular wall, lipid-based formulations like liposomes, liquid crystals, micelles are typically composed of natural phospholipids and cholesterol that have been used to deliver AcI with poor water solubility, which requires surfactant use, which can contribute to the toxicity of pesticides [36,39].

The characterization of biological safety of nanopesticides is an arduous task due to the complexity of nanostructures, reactivity, size, shape, and electric charge, making it difficult to assess, universalize and predict essential aspects of cytotoxicity and genotoxicity. The toxicity of glyphosate, a non-selective broad-spectrum organophosphate, has been of concern for manufacturers, workers, and food security. The glyphosate and its metabolite, aminomethylphosphonic acid, was found to be responsible for side effects on terrestrial and aquatic organisms. Allergic reactions, cardiac and respiratory problems, and endocrine disruption on human placental JEG3-cell line have seen in humans. The presence of albumin, surfactants, and solvents like 1,4-dioxane derivates increases nanoparticles’ toxicity [31,43,76,77,78].

Pesticides like clomazone herbicide, when sprayed, drift off crops by adjacent areas and expose animals and humans to dangerous toxicity risks. The effect of this herbicide has evaluated in free form and encapsulated in polymeric blend nanoparticles structured by ionic bound [20,79]. The hepatic effect of nanoparticles and clomazone in both forms to sublethal doses have evaluated in tadpoles (*L. catesbeianus*) submitted to acute exposure. According to results, there was an increase in the frequency of macrophage aggregates (melano-macrophage centers), eosinophils accumulation and lipidosis in the liver of experimental groups exposed to clomazone [79]. In this study, the polymeric nanoparticles blend did not increase the hepatic protective effect on tadpoles. In general, placental cytotoxicity leads to reproduction problems.

dsRNA directed against pest target genes gives protection against insect through plant-mediated RNAi as a strategy for insect pest control. Consequently, specific gene choice and dsRNA fragment design are of the highest potential to avoid cross toxicity with a non-selective target organism. However, this hypothesis, although intensely defended, has not been evaluated.

The RNA endogenous interference pathway’s specific sequence is efficient for genes target suppression and has enabling insecticides with an extended half-life; however, some gaps as a delivery mechanism, low stability in the environment, toxicity to non-target insects, risk of resistance by insects after chronic exposure to RNAi plants and the environmental destination has not been filled in [32,63]. After getting over these shortcomings, the RNAi-based products, in a short time, will revolutionize agricultural practice management and environmental protection effectively and safely.

Atrazine, intensely used in corn and sugarcane crops, is an environmental persistence pesticide, and due to its adverse impact on soil and aquatic ecosystems, it causes great concern. In Brazil, level nine times up at permitted (2 μg·L^−1^) has often registered in hydrographic basins [80,81]. The toxicity and potential risks of atrazine to aquatic fauna were investigated by the sensitivity measurement of Pacu (*P. mesopotamicus*) fingerlings for a dose of atrazine equivalent to LC50. The results showed that the liver was the organ with more severe damage. Although the number of micronuclei and nuclear abnormalities has remained unchanged, atrazine has induced cellular stress in the liver and kidney [80]. The potential toxicity of atrazine on Asian clam (*C. flumineausing*) was assessed, and the results showed that the atrazine affected the biotransformation mechanism and caused oxidative and DNA damage but did not present any relevant change in antioxidants defenses [82]. The genotoxic effect on *C. flumineausing* seems to occur by the chemical interaction of DNA with guanine and adenine bases through intercalation mechanism and adducts formation [82,83].

Imazethapyr is a nicotinic acid-derivate of imidazolinones herbicides group within Class III (slightly toxic agrochemical). However, it has recognized as a dangerous AcI for the environment and irritant for the eyes and the respiratory system. Insects and earthworms have different sensitivity to imazethapyr—bees and annelids are highly sensitive while earthworms have low or no sensitivity. The role genotoxicity induced by imazethapyr was measure by the oxidative DNA damage method [84]. The founds proved that imazethapyr (0.39–1.17 mg·L^−1^) induced nuclear abnormalities when tadpoles stayed exposed for 96 h with a significant increase of GDI (genetic damage index) after 48 h. The authors reported evidence of imazethapyr’s ability, spread across water or air, to endanger a risk to non-target exposed animal species [74,85]. Although imazethapyr is known for its pivotal role in weed control and the damage it causes to the ecosystem, no study to solve this paradox using nanostructured systems has been found in scientific publications.

Fipronil is a phenylpyrazole pesticide used for pest control in agriculture, veterinary and health care fields. Fipronil acts on γ-aminobutyric acid receptors of the insect, causing the depolarizing effect. It is declared more toxic to insects than to vertebrates; however, it causes side effects and alteration of the hepatic fat accumulation, reproductive cycle, DNA damage, and spermatozoa apoptosis have been reported. In vitro tests indicate that fipronil negatively affects mouse preimplantation embryos development, enhanced apoptosis, and reduces cell proliferation. In vivo tests have confirmed the in vitro observation and showed that fipronil is a risk factor in vertebrate reproduction [86]. The long-term exposure to toxic agrochemicals causes bioaccumulation of fatty acid in hepatic cells, leading to fibrosis and hepatitis [87]. The accumulation of ROS has a critical role in the hepatic and is responsible for inflammation in the liver cause hepatic tissue injuries [88].

## 6. Potential Ameliorative Impact of Nanoparticles

AcI–where bioavailability is limited by a low absorption rate—even though playing a pivotal role in controlling weeds, its use generates environmental problems given the necessity of the unusual doses. The AcI loaded in polymeric nanoparticles has been evaluated for environmental protection and weed control to solve the dose–effect paradox and enhance the safe level. The nanoparticulated system engineered with PLGA controlled the atrazine release, used as an AcI model, increased the bioavailability, and reduced the environmental toxicity level [75]. This finding is especially important because PLGA nanoparticles have shown the potential for atrazine-resistant crops.

The cerium oxide nanoparticles have been used clinically as an antioxidant in scavenging ROS, and cerium oxide nanoparticles have used to evaluate the protection level against hepatotoxicity and lipogenesis induced by fipronil. A study to examine the beneficial impact of fipronil-cerium oxide nanoparticles on hepatic tissue was evaluated in vivo. The results proved that cerium oxide nanoparticles had avoided the inflammation process in rats’ blood, reducing the bioaccumulation of fatty acids and reducing liver damage [87].

Metallic nanoparticles synthesis, with approaches GRAS, is relatively cheap and does not require chemically hazardous reducing agents. Metallic nanoparticles have large surface area properties, a comprehensive action spectrum against pathogens (bacterial and fungus), a negligible risk for resistance development, and low cost [23]. Unlike bulk metal homologues, metal nanoparticles are effective due to the antimicrobial biochemical mechanism explaining the metallic-nanoparticles actions [89]. Besides, metallic nanoparticles effectively control fungal and bacterial disease when used for immediate release in the crops [24]. However, reports about pulmonary inflammation [33] and strong immune system response have been attributed to CuNP coating with polysaccharides and copper nanoparticles exposure and bioaccumulation effect on living organisms [90].

A study conducted to assess the risks of six Cu-based nanomaterials reached a collection of results with a goal intended to supply a perspective on the risks of copper nanoparticles. The results showed that Ag-NP added in the soil are suddenly sulfidated in reducing environments such as freshwater sediment, decreasing the solubility and the bioavailability of sulfidized silver-nanoparticles (sAg-NP). Furthermore, cytotoxicity of sAg-NP was reduced, inferring that sulfidation would result in environmental decreased toxicity of Ag-NPs [25]. A study conducted to assess the risks of six copper-based nanomaterials reached a collection of results with a goal intended to supply a perspective on the risks of copper nanoparticles. The results showed that the production of ROS species and the nutrient imbalance due to the sequester of anionic mineral elements like phosphate, nitrate, and sulfate had a critical role in the toxicity mechanism [24]. The impact of sulfidation on dissolution, absorption, and toxicity of Ag-NP was studied. The results showed that Ag-NP added in the soil are suddenly sulfidated in reducing environments such as freshwater sediment, decreasing the solubility and the bioavailability of sAg-NP. Furthermore, cytotoxicity of sAg-NP was reduced, inferring that sulfidation would result in environmental decreased toxicity of Ag-NPs [25].

## 7. Conclusions

Agrochemical companies are focused on laying down the crop regardless of the safest condition and conventional pest management. However, it is necessary to recognize a great effort in research and financial support to find the best way to meet the agricultural demand for productivity and preserve human health and ecosystems.

The positive effects of nanotechnology in crops are well understood, while the potential toxicological effects and impacts of nanopesticides for environmental and food safety have received little attention. The short-term and long-term ACI adverse effects as a consequence of occupational or accidental ingestion of residues of pesticides from food, water-drinking and air are fatal or DALY due to permanent tissue and organ damage. Therefore, despite the efforts, the global toxic effects of nanopesticides and the toxicity mechanism are poorly understood. In evaluating the pros and cons, it is necessary to consider that the toxicity of nanopesticides may differ depending on the specific properties of the nanocarriers and AcI; there is, until the moment, a slight possibility for short-term changes. Therefore, the focus should be on nanoparticulate systems and their interaction with plants and the environment, highlighting reactivity, retention time, levels of bioaccumulation, biodegradation time, waste toxicity, and maxima reduction of leaching and drifting.

The rational design in the development of nanopesticides is limited by data scarcity, for example, determining which type of performance is expected for each type of nanopesticide after exposure. In most development studies involving in vivo assessment, it is not noticeable that the extrapolation of knowledge from one type of nanopesticide to another, especially concerning the interactions of nanocarriers with biological components (cell membrane, proteins, saccharides, enzymes) or physiological processes (absorption, biodistribution, metabolism, bioaccumulation, clearance), among other factors, is no less important. Functionalized nanoparticles are commonly used in pharmaceutical science to overcome limitations such as poor water solubility, chemical, physical and biological degradation, low permeation and bioavailability rate, absence of bioadhesion, immediate release, and fast biodistribution and side effects. Innovative formulations of nanopesticides offer many benefits and novel functions compared with older pesticide formulations; consequently, they also exhibit different properties in the environment.

Current perspectives reveal concerns about developing pathogens resistant to fungicides and other antimicrobials and show practical solutions to address this problem using pesticides based on nanoparticles with more than one antimicrobial mechanism of action and the development of target gene systems, respectively, metallic and bimetallic nanoparticles, and siRNA carried in functionalized IPEC. The new properties raise questions about the environmental disposition and fate of nanopesticides and their exposure to pollinators. The effect of pesticides on declining insect and aquatic invertebrates populations is an emergent global concern. Pollinating insects are responsible for three-quarters of crop pollination; therefore, they have an essential role in the agricultural ecosystem, particularly for humanity’s subsistence. The experimental research for solutions of inconveniences has been developing technology-based pesticides using diverse materials and techniques to prepare nanopesticides with modified release systems controlled by stimuli-responsive compounds that cover nanocarriers’ surfaces are able to remodel agricultural production while ensuring the preservation of ecosystems and food safety. This type of strategy based on environmental phenomena such as light, humidity, pH, and temperature can control the release time of pesticides, improve utilization efficiency, minimize short-term impacts, ensure pest control, decrease leaching, drift losses, and reduce environmental impact damage. Nonetheless, in the coming years, metallic nanoparticles may be pivotal for sustainable agriculture and preserving the ecosystem.

A growing trend towards risk classification in silico and statistical tools will contribute to setting up a predictive ecotoxicological pattern capable of estimating uncertain acute concentrations of AcI and predicting environmental damage in situ and toxicological effects in vivo. Therefore, the pathway to innovation in the crops requires an interdisciplinary approach which combines in silico tools, chemical and non-chemical pest control, engineering to develop innovative nanoparticles, auto-regulated systems, efficiency use, food safety, and small environmental implications.

## Figures and Tables

**Table 1:** Chemical and physical systems as carrier agents in nanopesticides formulation.

Chemical System				
***Covalent Bond***	***Carrier System***	***Formulation***	***Pesticides***	***Refs.***
***comonomers***	Hybrid materials	(CNT-g-PCA)	Zineb Mancozeb	[18]
***multifunctional system***	Peptide-polymer	Trypsin-PEG	Modulating oostatic factor	[19]
***Ionic bond***	Hallow sphere	Calcium-alginate	Cypermethrin	[20]
***Electrostatic complex***	Polyelectrolyte complex	Clay-gelatine pGPMA-dsRNA	MCPA dsRNA	[21,22]
***Cluster***	Metallic nanoparticles	Cu—TM Cu^0^, Ag^0^	Thiophanate methyl	[23,24,25]
**Physical System**				
***Encapsulation***	Coprecipitation Polycondensation Vesicle	Polyelectrolytic interaction. Cation vesicular surfactants	Trichlorfon Acetochlor Benzoylurea-paraquat DNA, RNA Copper	[12,26,27,28,29,30,31,32,33]
***Emulsion***	Mixed micelles Pickering emulsion Nanoemulsion Liquid crystal Liposome	mPEG13–b–PLGA5–3 Alginate-Ca++ Water-in-oil Monoolein 18-99 PC-chitosan	Pyrethrin γ-cyclodextrin Citronella Phytantriol α-cypermethrin	[34,35,36,37,38,39]
***Matrix system***	Hybrid materials	mPEG-PLGA	Metolachlor	[10,18]
***Porous system***	Grafted-NP. Sol-gel composite	4-ethylortho-Silicate ATP-biochar colloidal silica	Benzoylurea-Fe_2_O_3_ Glyphosate	[31,40,41,42,43]
***Foams***	Polymeric emulsion	Poly(alkylene-oxide] alkanol	Glyphosate acid Acetochlor	[29,44]
***Osmotic pumps***	Polymeric coating	Cellulose ester/ PEG/Inorganic salt	Diazinon	[45]

ATP (attapulgite); CNT-g-PCA (carbon nanotube-polycaprolactone); mPEG13–b–PLGA5–3 (monomethoxy (polyethylene glycol)13-poly(D, L-Lactide-co-glycolide); NP (nanoparticle); MCPA (4-chloro-2-methylphenoxy-acetic acid); PC (phosphatidyl choline) PEG (polyethylene-glycol); pGPMA (guanidine-propyl methacrylamide polymers); PLGA (poly-lactic-glycolic acid); dsRNA (double-stranded RNA).

**Table 2:** Innovative nanomaterials for stimuli-responsive release.

Mechanism/Nanomaterials	Pros Effect	Cons Effect	Refs.
**Depot**	Continuously release, utilization efficiency	Slow insect toxicity non-target	[48]
**Target**	Safe	High costs	[3,12]
**Stimuli-responsive** **[thermic, light, pH, ion,** **humidity,]**	Controlled release, reducing the loss, increased efficiency, biosensor, fast action, high availability	Random control Irreversible phase of AcI release, enhanced cellular uptake. Low selective toxicity, low biodegradability, induced pesticide resistance in target organisms	[12,42,43,50,51,52,53]
**Carbon nanotubes**	Biosensor; water uptake	Rise of ROS and cell death	[54]
**Polymer-protein conjugated**	Decrease bacterial resistance	Non-target	[19,21]
**Complex system polymer-based**	Increase bioavailability decrease leaching/drift. Catalytic reduction. Biosensor	Low environmental stability	[20,55]
**Interpolyectrolyte complex**	Multifunctional. Overcome multidrug resistance	Low chemical stability	[22]

## Data Availability

Not applicable.

## Abbreviations

**AcI**	**Agrochemical ingredients**
**ATP**	Attapulgite
**AgNP**	Silver nanoparticle
**AuNP**	Gold nanoparticle
**CuNP**	Cuprum nanoparticle
**CNT-g-PCA**	Carbon nanotube-polycaprolactone
**dsRNA**	Double-stranded RNA
**GRAS**	Generally recognized as safe
**IPEC**	Interpolyelectrolyte complex
**LDHs**	Layered double hydroxides
**MCPA**	4-chloro-2-methylphenoxy-acetic acid
**mPEG13–b–PLGA5–3**	Monomethoxy (polyethylene glycol)13-poly (D, L-Lactide-co-Glycolide)
**MWCNT**	Multi-walled carbon nanotube
**NP**	Nanoparticle
**PEG**	Polyethylene-glycol
**PC**	Phosphatidyl choline
**PE**	Pickering emulsion
**PEO-PMAA**	Poly-ethylene oxide-b-methacrylic acid
**pGPMA**	pGuanidine-propyl methacrylamide polymers
**PLGA**	Poly-lactic-glycolic acid
**siRNA**	Small interfering RNA
**α-RAFT**	Reversible addition-fragmentation chain transfer
